# The evaluation of dual-task conditions on static postural control in the older adults: a systematic review and meta-analysis protocol

**DOI:** 10.1186/s13643-019-1107-4

**Published:** 2019-07-27

**Authors:** Luca Petrigna, Ewan Thomas, Ambra Gentile, Antonio Paoli, Simona Pajaujiene, Antonio Palma, Antonino Bianco

**Affiliations:** 1 Program in Health Promotion and Cognitive Sciences, Department of Psychology, Educational Science and Human Movement, University of Palermo, Via Giovanni Pascoli 6, 90144 Palermo, Italy; 2 Department of Psychology, Educational Science and Human Movement, University of Palermo, Via Giovanni Pascoli 6, 90144 Palermo, Italy; 3 Department of Biomedical Sciences, University of Padova, Via F Marzolo 3, 35131 Padova, Italy; 4 Department of Coaching Science, Lithuanian Sports University, Sporto 6, LT-44221 Kaunas, Lithuania; 5 Regional Sport School of CONI Sicilia, Italian National Olympic Committee, Sicilia, Italy

**Keywords:** Multiple task, Aged, Postural balance, PRISMA-P, protocol, Review protocol

## Abstract

**Background:**

In postural stability evaluation, the dual-task concept is often adopted in order to create a more challenging situation. The dual-task consists of performing simultaneously two tasks, a primary static or dynamic motor task and an additional secondary cognitive task. Usually, a multitask condition leads to a reduction in the postural control performance, especially in older adults. Considering the wide spectrum of secondary task conditions existing in scientific literature, the present manuscript aims to write a peer-reviewed protocol that will be used in a systematic review and meta-analysis performed to identify the effects of different secondary tasks conditions in a population of older adults during static postural stability.

**Methods:**

The study will follow the Preferred Reporting Items for Systematic Reviews and Meta-Analyses (PRISMA) statement and for this manuscript, the PRISMA Protocol. PICOS criteria (population, intervention, comparison, outcomes, study design) will be also followed. The population examined will be healthy older adults over 60 years of age and all quantitative, qualitative, and mixed methods study design will be included. Original articles will be also included if written in English, while no restriction criteria will be applied to the country of origin. Instead, reviews, meta-analysis, abstracts, citations, scientific conferences, opinion pieces, books, books reviews, statements, letters, editorials, non-peer reviewed journals articles, and commentaries will be excluded. The research of literature will be performed using PubMed, Web of Science, and Scopus with words related to the topic. From each included study, information previously agreed will be extracted and inserted into a spreadsheet and a narrative synthesis containing summary tables and graphs will describe the articles taken in examination. Furthermore, a meta-analysis will be performed to establish which DT condition has a greater effect following the Hedges and Olkin approach, extension of Glass’ method and Cohen’s *d* will be calculated.

**Discussion:**

The present manuscript wants to provide the protocol that will be used in the systematic review and meta-analysis with the intent to inform the researchers and professionals about the dual-task condition effects. Such will lead future investigations in using the most appropriate dual-task condition.

**Systematic review registration:**

PROSPERO CRD42018116597.

## Background

The dual-task (DT) condition involves the performance of two tasks performed concurrently, a primary static or dynamic motor and a secondary cognitive task [1]. Such conditions compromise postural stability especially in older adults, due to deterioration in both the postural control system and the ability to allocate attention [2–4] because especially in this population, postural control seems to be an attention-demanding task [5]. Furthermore, the increase in attentional-demand [6] corresponds to a decrease in postural stability when the complexity of a postural or cognitive task is increased [2, 4, 7].

Even if most of the DT conditions involve the use of working memory [8], there are several studies that use a wide variety of DT conditions such as carrying an object [4] or tapping the fingers [9] which are named manual. Between the cognitive tasks, there are visual-verbal tasks [10] or verbal-only tasks [11] or auditory tasks [3]. Other secondary tasks can impose attentional demands involving manual [12], visual [13], auditory [14], or reaction time [15] tasks. To summarize, the DT conditions can be classified as manual, reaction time, discrimination and decision making, mental tracking, verbal fluency, and working memory tasks [8, 16]. This wide spectrum of DT conditions contributes to increase the complexity in the evaluation of postural stability [17] which could generate confusion. In a review and meta-analysis of Ghai et al. [18], the consequence of different DT conditions on training programs have been evaluated; however, further evaluation through a systematic review and meta-analysis may better clarify the effect that each different secondary task has on postural stability in older adults.

The first step for high-quality research level is the publication of the protocol with a rigorous methodological design, is peer-reviewed [19], is established before the review of literature, and reports and explains any change made after the publication of the protocol [20]. For these reasons, the present manuscript aims to describe the protocol in order to standardize the research methodology to evaluate the effects of different secondary tasks and to determine which is the most challenging through a systematic review of the literature and meta-analysis.

## Methods/design

The protocol will follow the Preferred Reporting Items for Systematic Reviews and Meta-Analyses (PRISMA) statement [21] and for this manuscript, the PRISMA Protocols (PRISMA-P) [22]. When authors change the protocol, these are documented and published in the final work. The protocol for the systematic review has been registered in PROSPERO (http://www.crd.york.ac.uk/PROSPERO/display_record.php?ID=CRD42018116597), registration number is CRD42018116597.

### Eligibility criteria

The authors choose to follow the PICOS criteria (population, intervention, comparison, outcomes, study design) described in PRISMA [21] (Table 1).

**Table 1:** Inclusion and exclusion criteria according PICOS guidelines (population, intervention, comparison, outcomes, study design)

PICOS	Inclusion criteria	Exclusion criteria
Population	Healthy adults older than 60 years of age.	Mental, psychological, physical, or motor disorders that may influence postural control.
Intervention	Intervention or comparison with a dual-task condition in static postural balance.	Interventions or comparisons that present dynamic postural balance.
Comparison	The comparison will be between single-task and dual-task condition.	
Outcomes	Dual-task conditions to study static postural control.	
Study design	All quantitative, qualitative, and mixed-methods designs.	Reviews, meta-analysis, abstracts, citations, scientific conference abstracts, opinion pieces, books, book reviews, statements, letters, editorials, non-peer reviewed journal articles, and commentaries in the review.

The included population comprised adults of both genders who were older than 60 years of age. Exclusion criteria were mental (i.e. neurodegenerative disease, intellectual disability, psychosis or obsessive-compulsive disorders), psychological (i.e. personality disorder, somatic symptom disorder, dementia or delirium), physical (i.e. muscular dystrophy, hypotonia, injury to tendons or ligaments), or motor disorders (i.e. akinesia, bradykinesia or dystonia) that may influence postural stability and not due to the physiological decline of advancing age.

Any type of intervention will be included if the protocol consists of the following: (1) the evaluation of static postural stability while performing a secondary task condition, (2) participants performed the postural stability task with parallel feet, (3) the test was performed on a firm surface because of a greater reliability of the data [23], and (4) with eyes open to avoid the effect on centre of pressure measures that the removed visual feedback causes [24]. Instead, all kinds of interventions that consist of the performance of a dynamic primary task condition will be excluded.

The comparison will be considered between postural stability measured in a single static task and during the DT condition.

The outcomes will summarize the use of DT conditions to study postural stability when older adults are standing in a static upright posture. Regarding the typology of data, there are different centre of pressure measures such as planar deviation (mm), anteroposterior range or mediolateral range (mm), mean distance (mm), root mean square distance (mm), confidence circle area (mm^2^), confidence ellipse area (mm^2^), mean velocity (mm/s), mean frequency (Hz), phase plane parameter, fractal dimension, critical time interval, mean squared critical displacement, diffusion constant, but only a few of them (root mean square distance, mean velocity, mean frequency, phase plane parameter, and fractal dimension) present acceptable levels of relative and absolute reliability [25]. The major limitation is the different levels of reliability of the centre of pressure measures [26] and also the wide spectrum of units of measure. For this reason, only the distance (centre of pressure sway) and area of sway will be taken into consideration.

For the study design, all quantitative, qualitative, and mixed-methods designs will be included. Reviews, meta-analysis, abstracts, citations, scientific conference abstracts, opinion pieces, books, book reviews, statements, letters, editorials, non-peer reviewed journal articles, and commentaries in the review will not be include in the systematic review and meta-analysis.

### Information sources

The sources of information include PubMed, Web of Science, and Scopus. Data screening will be performed for studies published on the title and topic of DT conditions without any time restriction (the end date will be the date on which the article search will be completed).

### Search strategy

The following groups of search terms will be used in combination with the Boolean indicator AND:

- Keywords1: “dual task*”, “double task*”, “multiple task*”, “secondary task*”, “second task*”, “two task*”, “double assignment*”, “cognitive task*”.
- Keywords2: “postural control*”, “postural stability*”, “postural balance*”, “postural equilibrium*”, “postural sway*”, “posturography*”, “stabilometry*”, “baropodometry*”.
- Keywords3: “elderly”, “old adult*”, “senior*”, “aging”, “ageing”, “aged”, “ancient”.

Each search term will be used in combination with another search term of other groups and the following in a string example that will be used in PubMed, Web of Science, or Scopus: ((“dual task*” OR “double task*” OR “multiple task*” OR “secondary task*” OR “second task*” OR “two task*” OR “double assignment*” OR “cognitive task*”) AND (“postural control*” OR “postural stability*” OR “postural balance*” OR “postural equilibrium*” OR “postural sway*” OR “posturography*” OR “stabilometry*” OR “baropodometry*”) AND (“elderly” OR “old adult*” OR “senior*” OR “aging” OR “ageing” OR “aged” OR “ancient”)).

### Study record

#### Data management

Manuscript collection will be performed by two authors (LP and ET) working independently. Afterward, the manuscripts selection will involve a two-step process: a first selection using the function “find duplicates” included in EndNote (EndNote version X8; Thompson Reuters, New York, USA) for a computer-automated selection process; in a second moment, a manual review will be done to identify appropriate manuscripts. The manual selection will involve two investigators (LP and ET) who will work independently in a three-stage process. In the first stage, the titles of the selected manuscripts will be examined and screened against the eligibility criteria. In the second stage, the abstracts will be examined to identify eligible manuscripts. In the third stage, the full texts of the articles that appear to meet the inclusion criteria will be reviewed. In the case of disagreements between the investigators, a third investigator will consider the works independently and discuss the decision with the other investigators (AB and AP). The investigators will not be blinded to the manuscripts, study title, authors, or associated institutions during the screening process. A PRISMA flow diagram will be presented in the final output to summarize the search and screening processes.

#### Selection process

Manuscripts written in English will be considered irrespective of the country of origin. The selection criteria will comprise two parts: acquisition of the manuscript and then comparison according to the inclusion and exclusion criteria.

#### Data collection process

The data will be collected according to the present standardized protocol process. The following information will be extracted from each included study and inserted into a Microsoft Excel (Microsoft Corp, Redmond, Washington) spreadsheet: lead author, year of publication, type of study (randomized, non-randomized, intervention, non-intervention), sample size, participants’ age (range and mean ± standard deviation), sex, cultural background, education and physical or sport activities practised, objective of the study, single task technique, DT technique, and influence of DT condition relative to the single task. The relevant information will be extracted from any section of the manuscript.

The data for the meta-analysis will be collected from the results section and tables of the manuscripts. Eventually, the graphs will be also used to extrapolate data using the program WebPlotDigitizer (version 4.2). If it will be impossible to collect the data from the manuscript, the corresponding author of the manuscript will be contacted twice before the exclusion of the study from the meta-analysis.

### Risk of bias in individual studies

There are six domains for a potential risk of bias: selection bias, performance bias, detection bias, reporting bias, attrition bias, and unexplained bias [27]. Unfortunately, neither the Cochrane Risk of Bias tools [27] for randomized studies nor the ACROBAT-NRSI guidelines [28] for non-randomized trials can be used as the authors of this systematic review expect to find, collect, and analyse both, randomized and non-randomized studies. Furthermore, intervention and non-intervention studies will be taken into consideration.

Regarding the reporting bias, two different investigators (LP and ET) will subjectively evaluate the results of the article took in examination and if there may be a lack of data, the main author of the article will be contacted to obtain information. In the absence of an answer, the article will be excluded. Since the present systematic review and meta-analysis will not evaluate the effect of an intervention, the dropout or the differences between the groups will not influence the results (attrition bias).

Regarding the meta-analysis, the risk of publication bias will be detected through a visual and a geometrical appreciation of the studies. Specifically, a funnel plot will be created to visually detect the existence of the file drawer problem, while the geometrical evaluation will be conducted through the Egger regression test [29], assessing the asymmetry of the funnel plot.

### Data synthesis

All the data will be collected and summarized descriptively with tables and graphs, and they will be analysed through a descriptive narrative synthesis to determine the influence of specific DT conditions on static postural stability.

To evaluate the influence of the DT conditions, a meta-analysis will be performed by an expert (AG) and an investigator (ET) will follow the process. The package *metafor* of the R software (version 3.5.1) will be used to calculate the mean effect size given by the subtraction of the DT from the single-task performance. For each study, all the variables will be treated as continuous and mean and standard deviation will be recorded, while for those studies revealing mean and standard deviations within plots the effect size will be calculated with WebPlotDigitizer (version 4.2).

The meta-analysis will be conducted in accordance with the Hedges and Olkin [30] approach, an extension of Glass’ [31] method, with a significance level of *α* = .05. The heterogeneity across the studies will be assessed through the Cochrane’s Q and if the studies show high internal variability (thus, *p* < 0.05), a subgroup analysis will be performed comparing the DT conditions grouped according to the classification used by Al-Yahya et al. [16]: (1) manual task, (2) reaction time task, (3) discrimination and decision-making task, (4) mental tracking task, (5) verbal fluency task, (6) working memory groups, and (7) an additional category named “other” that will include different DT conditions that do not belong to the previously named groups.

## Discussion

This protocol review would be a solid starting point that will allow to perform a systematic review and meta-analysis to examine the influence of different DT conditions on static postural stability in older adults. The strength in publishing the protocol before performing the systematic review and meta-analysis is that this is evaluated and revised with a “peer-review” system to improve its quality, even if one possible limitation could be the lengthening of the publication time of the manuscript [32].

The need to study this topic arises because, according to the scientific literature [7], the secondary task should create the conditions making the primary task measurable through achieving a sufficient difficulty in order to reach or exceed the neural resource limits. The second point highlighted by Boisgontier et al. [7] is that the DT condition should involve the association between brain areas and in addition to influence the intrinsic difficulty of the main task performance using a generic interference. Finding the effect size of different DT conditions will help future investigators to use the most appropriate DT during the experiments and allow trainers to create an appropriate intervention to prevent falls. Furthermore, the aim to identify the effect of different DT conditions in older adults is important to improve the knowledge in intervention protocols to effectively engage higher cognitive and physiological adaptations.

One possible limitation will be the control of the risk of bias. First, regarding the selection bias, the population that will be taken into consideration will be composed by healthy older adults, but the postural stability during DT is influenced by intelligent quotient of the participants, in the tasks where reading and executing math computation performance is required [33]. Another possible limitation is the inability to validate not only the homogeneity of the participant, participants’ backgrounds and transient attentional factors [12, 34], and anxiety level [35] but also disorders not detected during the screening of each study as vestibular, vision, and hearing disorders which may also influence the DT outcomes.

Another factor that needs to be taken into consideration is the comparison between tasks performed silently and those in which it is required to speak, because these latter tasks could be influenced by one’s respiration [36]. Also, the visual stimuli, that can be present in some tasks, may influence the sway oscillation pattern [37]. Another limitation related to the risk of bias could be due to the different parameters (root mean square instead of area or the centre of pressure or velocity) that are usually used during the postural stability data collection. Finally, the different duration of the trials and therefore the recording time used in the manuscripts, could influence the results [38] and consequently interfere during the performance of the meta-analysis. The investigators of the present manuscript plan to overcome these differences by dividing the tool sample into multiple groups and making separate analyses. To conclude, there are different causes which may influence the protocol, for this reason, to guarantee a high-level manuscript, it is important to have a peer-reviewed protocol before the future systematic review and meta-analysis.

## Data Availability

Not applicable

## Abbreviation

DT: dual-task

## References

[1] Woollacott M, Shumway-Cook A (2002). Attention and the control of posture and gait: a review of an emerging area of research. Gait Posture.

[2] Huxhold O, Li SC, Schmiedek F, Lindenberger U (2006). Dual-tasking postural control: aging and the effects of cognitive demand in conjunction with focus of attention. Brain Res Bull.

[3] Shumway-Cook A, Woollacott M (2000). Attentional demands and postural control: the effect of sensory context. J Gerontol A Biol Sci Med Sci.

[4] Granacher U, Bridenbaugh SA, Muehlbauer T, Wehrle A, Kressig RW (2011). Age-related effects on postural control under multi-task conditions. Gerontology..

[5] Brown LA, Shumway-Cook A, Woollacott MH (1999). Attentional demands and postural recovery: the effects of aging. J Gerontol A Biol..

[6] Woollacott MH (2000). Systems contributing to balance disorders in older adults. J Gerontol A Biol.

[7] Boisgontier MP, Beets IA, Duysens J, Nieuwboer A, Krampe RT, Swinnen SP (2013). Age-related differences in attentional cost associated with postural dual tasks: increased recruitment of generic cognitive resources in older adults. Neurosci Biobehav Rev.

[8] Funahashi Shintaro (2017). Working Memory in the Prefrontal Cortex. Brain Sciences.

[9] Chagdes JR, Rietdyk S, Haddad JM, Zelaznik HN, Raman A, Rhea CK (2009). Multiple timescales in postural dynamics associated with vision and a secondary task are revealed by wavelet analysis. Exp Brain Res.

[10] Bonnet CT, Baudry S (2016). Active vision task and postural control in healthy, young adults: synergy and probably not duality. Gait Posture..

[11] Coelho T, Fernandes A, Santos R, Paul C, Fernandes L (2016). Quality of standing balance in community-dwelling elderly: age-related differences in single and dual task conditions. Arch Gerontol Geriat.

[12] Pajala S, Era P, Koskenvuo M, Kaprio J, Tolvanen A, Rantanen T (2007). Genetic and environmental contribution to postural balance of older women in single and dual task situations. Neurobiol Aging.

[13] Redfern MS, Jennings JR, Martin C, Furman JM (2001). Attention influences sensory integration for postural control in older adults. Gait Posture.

[14] Marsh AP, Geel SE (2000). The effect of age on the attentional demands of postural control. Gait Posture.

[15] Logan Gordon D., Schachar Russell J., Tannock Rosemary (1997). Impulsivity and Inhibitory Control. Psychological Science.

[16] Al-Yahya E, Dawes H, Smith L, Dennis A, Howells K, Cockburn J (2011). Cognitive motor interference while walking: a systematic review and meta-analysis. Neurosci Biobehav R.

[17] Dault MC, Yardley L, Frank JS (2003). Does articulation contribute to modifications of postural control during dual-task paradigms?. Cognitive Brain Res.

[18] Ghai S, Ghai I, Effenberg AO (2017). Effects of dual tasks and dual-task training on postural stability: a systematic review and meta-analysis. Clin Interv Aging.

[19] Klugar M (2016). A protocol is essential for a systematic review as randomization is for randomized controlled trials. JBI Database System Rev Implement Rep.

[20] Koensgen N, Rombey T, Allers K, Mathes T, Hoffmann F, Pieper D (2019). Comparison of non-Cochrane systematic reviews and their published protocols: differences occurred frequently but were seldom explained. J Clin Epidemiol.

[21] Moher D, Liberati A, Tetzlaff J, Altman DG, Group P (2009). Preferred reporting items for systematic reviews and meta-analyses: the PRISMA statement. Open Med.

[22] Moher D, Shamseer L, Clarke M, Ghersi D, Liberati A, Petticrew M (2015). Preferred reporting items for systematic review and meta-analysis protocols (PRISMA-P) 2015 statement. Syst Rev.

[23] Ruhe A, Fejer R, Walker B (2010). The test-retest reliability of centre of pressure measures in bipedal static task conditions - a systematic review of the literature. Gait Posture..

[24] Blaszczyk JW, Prince F, Raiche M, Hebert R (2000). Effect of ageing and vision on limb load asymmetry during quiet stance. J Biomech.

[25] Qiu H, Xiong S (2015). Center-of-pressure based postural sway measures: reliability and ability to distinguish between age, fear of falling and fall history. Int J Ind Ergon.

[26] Lin DD, Seol H, Nussbaum MA, Madigan ML (2008). Reliability of COP-based postural sway measures and age-related differences. Gait Posture..

[27] Higgins JP, Altman DG, Gotzsche PC, Juni P, Moher D, Oxman AD (2011). The Cochrane Collaboration's tool for assessing risk of bias in randomised trials. BMJ..

[28] Sterne JAC, Hernan MA, Reeves BC, Savovic J, Berkman ND, Viswanathan M, et al. ROBINS-I: a tool for assessing risk of bias in non-randomised studies of interventions. BMJ. 2016;355:i4919. 10.1136/bmj.i4919. 10.1136/bmj.i4919 PMC5062054 27733354

[29] Egger M, Smith GD, Schneider M, Minder C (1997). Bias in meta-analysis detected by a simple, graphical test. BMJ.

[30] Hedges Larry V., Olkin Ingram (1985). Estimation of a Single Effect Size: Parametric and Nonparametric Methods. Statistical Methods for Meta-Analysis.

[31] Glass GV (1976). Primary, secondary, and meta-analysis of research. Educ Res.

[32] Allers K, Hoffmann F, Mathes T, Pieper D (2018). Systematic reviews with published protocols compared to those without: more effort, older search. J Clin Epidemiol.

[33] Mayes SD, Calhoun SL, Bixler EO, Zimmerman DN (2009). IQ and neuropsychological predictors of academic achievement. Learn Individ Differ.

[34] Stins JF, Michielsen ME, Roerdink M, Beek PJ (2009). Sway regularity reflects attentional involvement in postural control: effects of expertise, vision and cognition. Gait Posture.

[35] Hadjistavropoulos T, Carleton RN, Delbaere K, Barden J, Zwakhalen S, Fitzgerald B (2012). The relationship of fear of falling and balance confidence with balance and dual tasking performance. Psychol Aging.

[36] Hodges PW, Gurfinkel VS, Brumagne S, Smith TC, Cordo PC (2002). Coexistence of stability and mobility in postural control: evidence from postural compensation for respiration. Exp Brain Res.

[37] Richer N, Lajoie Y. Cognitive task modality influences postural control during quiet standing in healthy older adults. Aging Clin Exp Res. 2018:1–6. 10.1007/s40520-018-1068-9. 10.1007/s40520-018-1068-9 30414089

[38] Raymakers JA, Samson MM, Verhaar HJJ (2005). The assessment of body sway and the choice of the stability parameter(s). Gait Posture.

